# Synergy of Losartan and chemotherapy for patients with cholangiocarcinoma: A propensity score-matched analysis

**DOI:** 10.3389/fonc.2022.989080

**Published:** 2022-11-23

**Authors:** Qing Li, Zhenyu Chang, Tianyi Wang, Bing Liu, Ximin Wang, Xin-Yu Ge, Tao Yang, Qu Liu, Wei Wang

**Affiliations:** ^1^ Department of Internal Medicine, Third Affiliated Hospital of Jinzhou Medical University, Jinzhou, China; ^2^ Department of Organ Transplantation, the Third Medical Center of Chinese People’s Liberation Army (PLA) General Hospital, Beijing, China; ^3^ Faculty of Hepatopancreatobiliary Surgery, the First Medical Center of Chinese People’s Liberation Army (PLA) General Hospital, Beijing, China; ^4^ Department of General Surgery, First Affiliated Hospital of Jinzhou Medical University, Jinzhou, China; ^5^ Department of Disease Control and Prevention, Rocket Force Characteristic Medical Center, Beijing, China

**Keywords:** Cholangio carcinoma, prognosis, PSM, losartan, chemotherapy

## Abstract

**Background:**

Cholangiocarcinoma (CCA) is a malignant tumor originating from bile duct epithelial cells that no obvious clinical symptoms and specific clinical manifestations are shown in the early stage of CCA.

**Methods:**

Propensity score matching (PSM) is a quasi-experimental method in which this study used. Patients were enrolled from Department of General surgery, First Affiliated Hospital of Jinzhou Medical University from March 1, 2010, to December 30, 2019. Totally 170 patients with CCA were enrolled in this study.

**Results:**

We performed a 1:2 PSM study and found that patients with losartan group showed both comparable median OS (overall survival) and TTR (time to recurrence) to those in the patients without losartan group before PSM. However, after matching, patients with losartan group showed favorable median OS and TTR than those in the patients without losartan group. Then we performed Cox proportional hazards models and found that patients with losartan was an independent factor after multivariable analysis for patients with CCA. Furtherly, we sequenced serial cfDNA were performed in 10 patients with losartan and 9 patients without losartan who received adjuvant chemotherapy after tumor resection. These results showed that the treatment of losartan was related with tumor microenvironment and could be potentially useful to combine the immunotherapy for patients with CCA.

**Conclusion:**

In conclusion, this study demonstrated that the treatment of losartan could increase the efficacy of adjuvant chemotherapy and identified as an independent survival predictor for patients with CCA. Moreover, losartan could be potentially useful to combine the immunotherapy for patients with CCA.

## Introduction

Cholangiocarcinoma (CCA) is a malignant tumor originating from bile duct epithelial cells that no obvious clinical symptoms and specific clinical manifestations are shown in the early stage of CCA (1, 2). The tumor is usually discovered due to the symptom of biliary tract obstruction in the later stage of the disease, at which point the optimal time for surgery has passed. As a result, the treatment effect and clinical prognosis of CCA are not satisfactory. Patients with surgically resected tumors have a 5-year survival rate of 5%, and the survival time of advanced patients who do not receive surgery is less than 1 year. At present, Cholangiocarcinoma is the second most common hepatic malignancy after hepatocellular carcinoma (HCC), and the overall incidence of cholangiocarcinoma has increased progressively worldwide over the past four decades. cholangiocarcinomas are categorized according to anatomical location as intrahepatic (iCCA), perihilar (pCCA), or distal (dCCA). Each subtype has a distinct epidemiology, biology, prognosis, and strategy for clinical management (3–5).

Surgery is the preferred treatment option for all three disease subtypes, but a minority of patients (approximately 35%) have early-stage disease that is amenable to surgical resection with curative intent. For patients with advanced-stage or unresectable cholangiocarcinoma, the available systemic therapies are of limited effectiveness: the median overall survival with the current standard-of-care chemotherapy regimen (gemcitabine and cisplatin) is <1 year. The combination of gemcitabine and cisplatin is the current first-line chemotherapy for patients with advanced-stage cholangiocarcinoma not amenable to locoregional and surgical options, irrespective of anatomical disease subtype. There are now 2 published randomized studies for iCCA using adjuvant chemotherapy (6–9). The adjuvant standard of care for all CCA subtypes has been established as capecitabine based on the BilCap study and reiterated in the NCCN guidelines,12 and as such patients with CCA treated with capecitabine as adjuvant therapy may anticipate a survival of 51.1 months. The PRODIGE12 study of gemcitabine plus oxaliplatin (GEMOX) in an adjuvant setting was clearly negative; perhaps because it was underpowered for outcome prediction. The iCCA subgroups of BilCap (n = 84) and PRO- DIGE12 (n = 86) had hazard ratios (HRs) of 0.65 (95% CI 0.35–1.18) and 0.718 (95% CI 0.431 to 1.196) respectively, although neither reached statistical significance. It must be emphasized that these *post hoc* analyses are exploratory rather than conclusive, particularly as the primary endpoint of the PRODIGE12 study was negative (10, 11).

In recent years, studies have suggested that angiotensin II (Ang II) signaling pathway plays an important role in tumor progression. Angiotensin II type 1 receptor (AT1R) overexpression is also associated with poor prognosis, and the Ang II/AT1R axis is closely related to tumor progression (12–14); however, there is also the opposite view. Renin-angiotensin system (RAS) is far more complicated than we think. It has a close relationship with the proliferation, angiogenesis, invasion and metastasis of tumor cells, especially in recent years, some new RAS components have further verified the role of RAS in the development of cancers. Angiotensin II, as the main vasoactive peptide of RAS, plays an important role in the treatment of kidney protection, reducing uric acid, improving insulin resistance, promoting skeletal muscle remodeling, delaying and reversing tissue organ fibrosis, while there is still controversy in tumor treatment. Losartan is an FDA-approved antihypertensive agent that blocks AGTR1 (15–17). Several studies have reported the synergy of losartan with chemotherapy across numerous tumors (15, 18–20). However, no report investigated the effect of losartan on chemotherapy for patients with CCA, which is the overarching objective of this study

In this study, we aimed to compare the survival outcomes related with the effect of losartan in patients CCA after surgery. Furthermore, we sought to assess the synergistic effect of losartan with chemotherapy in patients with CCA.

## Patients and methods

### Patients and treatment schedule

The study was approved by the Ethics Committee of the Institutional Review Board of the Department of General surgery, First Affiliated Hospital of Jinzhou Medical University. Written informed consent was waived owing to the retrospective and deidentified nature of the study. Between March 1, 2010, to December 30, 2019, patients who were pathologically diagnosed with cholangiocarcinoma and treated with curative resection and anti-cancers adjuvant chemotherapy agents as a first-line therapy in First Affiliated Hospital of Jinzhou Medical University were enrolled in this retrospective study. This study was conducted according to the principles of the Declaration of Helsinki. The clinical parameters were collected, including sex, age, stage, presence of liver/brain metastasis, diseases history, previous treatment lines and regimens, history of other treatments and laboratory data.

Next, Postoperative outcomes and treatment included the occurrence of major morbidity (Clavien-Dindo ≥ III) and 30-day mortality. Pathological parameters were collected according to the 8th edition of the AJCC staging system and included tumor stage, tumor size, extent of lymph node involvement, and tumor grading (21, 22).. Follow-up data for all patients were obtained from their most recent medical review, including documented clinical examination and assessment of computed tomography (CT) scans. Patients’ overall survival (OS) was calculated from the date of the index operation to the date of death or last contact. An independent biostatistician managed and maintained the collected data.

### Propensity score matching

Propensity score matching (PSM) is a quasi-experimental method in which the researcher uses statistical techniques to construct an artificial control group by matching each treated unit with a non-treated unit of similar characteristics. Using these matches, the researcher can estimate the impact of an intervention. In this study, Firstly, patients in groups with treatment of losartan and without treatment of losartan were paired by the propensity score method referred by Rubin and Rosenbaum and these process were completed by SPSS.22.0 software. The next process is the pairs removed from matching and then the other case was selected like previous step (23, 24).

### Identification of somatic alterations in patients

Plasma samples were collected before initial treatment and three months after initiating the DC-CIK. Next generation sequencing was performed on peripheral blood cell-free DNA (cfDNA) by a commercial vendor. Targeted sequencing was performed in 60 plasma cell-free DNA (cfDNA), as well as 30 germ line DNA samples. The target region is about 1.1Mb, which include coding exons and selected introns of 1021 genes. A total of 1021 genes were selected from four sources: 1) known oncogenes and tumor suppressor genes; 2) genes that are targets of agents approved by the FDA or have been assessed in clinical trials; 3) genes implicated in major cancer-related signaling pathways; 4) genes identified in the findings of the TCGA network which covers 12 cancer types. Sequencing libraries were prepared from ctDNA using KAPA DNA Library Preparation Kits (Kapa Biosystems, Inc.), and gDNA sequencing libraries were prepared using the protocols recommended by the Illumina TruSeq DNA Library Preparation Kit. For samples close to the minimum input requirement, additional pre-capture PCR cycles were performed to generate sufficient PCR product for hybridization. Libraries were hybridized to custom-designed biotinylated oligonucleotide probes (Integrated DNA Technology, Coralville, USA) covering target region sequence. DNA sequencing was carried out with the HiSeq3000 Sequencing System (Illumina, San Diego, CA). Somatic SNVs and InDels were detected using the Mutect 2.0 algorithm (https://software.broadinstitute.org/gatk/gatkdocs/current/org_broadinstitute_gatk_tools_walkers_cancer_m2_MuTect2.php) Somatic copy number alterations and structure variations were analyzed using local algorithms.

### Statistical methods

Continuous variables were expressed as mean ± SD (standard deviation) and compared using a two-tailed unpaired Student’s t test; categorical variables were compared using χ2 or Fisher analysis. The Greenwood formula was used for the standard deviation. A Cox regression approach was chosen for the evaluation of factors related with overall survival (OS) and time to recurrence (TTR) for patients with CCA (25). Results are reported as odd ratios (OR) and their 95% confidence intervals (CI). A OR > 1 indicated an elevated relation with respect to the reference category. A confidence interval which did not include the value 1 indicated statistical significance at the 5% level. All statistical evaluations were carried out using SPSS software (Statistical Package for the Social Science, version 15.0, SPSS Inc, Chicago, IL) and GraphPad Prism 5 (Version 5.01, GraphPad Software, Inc., USA). A value of p<0.05 was considered to be statistically significant in all the analyses.

## Results

### Patient characteristics

Patients were enrolled from Department of General surgery, First Affiliated Hospital of Jinzhou Medical University from March 1, 2010, to December 30, 2019. Totally 170 patients with CCA were enrolled in this study. After checking all the records in hospital, patients were allocated into two groups: patients with losartan years(n=34) and patients without losartan (n=136). Most patients received long term treatment of losartan because of their cardiovascular diseases and all patients with losartan included in this study at least received treatment of losartan for 4 weeks before and after the adjuvant chemotherapy. Characteristics of all patients are detailed in **Table 1**. There were significant differences in BMI, Preoperative AFP, hypertension, and the proportion of nodal status between these two groups before PSM. After the propensity score match (we used 1:2 matching since the limited number of patients with losartan), the cohort consisted of 30 patients with losartan and 60 compared patients without losartan. The clinical variables that could be obtained at the time of initial diagnosis and were considered to have influenced the outcomes were used for the 1:2 matching between the two groups. The standardized differences after matching were smaller for all background variables compared with those in the unmatched groups and no significant differences were shown between the two groups except for the hypertension status (**Table 2**).

**Table 1:** Patient and tumor characteristics before PSM.

Variable	without losartan (136)	with losartan (34)	p value
**Sex**			0.757
Male	76	20	
Female	60	14	
**Age (years)**	63.2 (35-79)	62.6 (34-78)	0.495
**BMI kg/m2**	25.8 ± 6.2	26.5 ± 4.9	0.014
**Serum CA-19-9 U/ml**			0.938
<37	57	14	
>37	79	20	
Preoperative AFP (μg/l)			0.012
<9	31	15	
>9	105	19	
**hypertension**			0.001
**yes**	27	34	
**no**	109	0	
**Tumor and pathologic characteristics**			
**AJCC TNM stage**			0.331
I	85	17	
II	37	11	
III	14	6	
**Tumor size(cm)**	2.47 ± 1.83	2.54 ± 1.74	0.128
**tumor number**			0.155
**single**	125	30	
**multiple**	11	4	
**Neural Invasion**			0.802
Yes	65	15	
No	71	19	
**Vascular invasion**			0.588
Yes	57	16	
No	79	18	
**Nodal status**			0.013
0	70	8	
1	46	19	
2	20	7	
**Margin invasion**			0.269
yes	28	10	
no	108	24	
**Tumor recurrence**			0.531
absent	45	8	
present	91	26	

**Table 2:** Patient and tumor characteristics after PSM.

Variable	without losartan (60)	with losartan (30)	p value
**Sex**			0.873
Male	31	20	
Female	28	14	
**Age (years)**	61.5 (38-77)	62.1 (34-74)	0.431
**BMI kg/m2**	24.7 ± 5.7	25.2 ± 5.4	0.375
**Serum CA-19-9 U/ml**			0.881
<37	27	14	
>37	33	16	
Preoperative AFP (μg/l)			0.827
<9	23	11	
>9	37	19	
**hypertension**			0.001
**yes**	12	30	
**no**	48	0	
**Tumor and pathologic characteristics**			
**AJCC TNM stage**			0.683
I	32	16	
II	24	11	
III	4	3	
**Tumor size(cm)**	2.35 ± 1.76	2.44 ± 1.52	0.574
**tumor number**			0.761
**single**	51	26	
**multiple**	9	4	
**Neural Invasion**			0.746
Yes	26	14	
No	34	16	
**Vascular invasion**			0.881
Yes	27	14	
No	33	16	
**Nodal status**			0.652
0	15	7	
1	35	17	
2	10	6	
**Margin invasion**			0.741
yes	22	10	
no	38	20	
**Tumor recurrence**			0.862
absent	15	7	
present	45	23	

### Survival analysis of patients with different treatments

**Figure 1:**
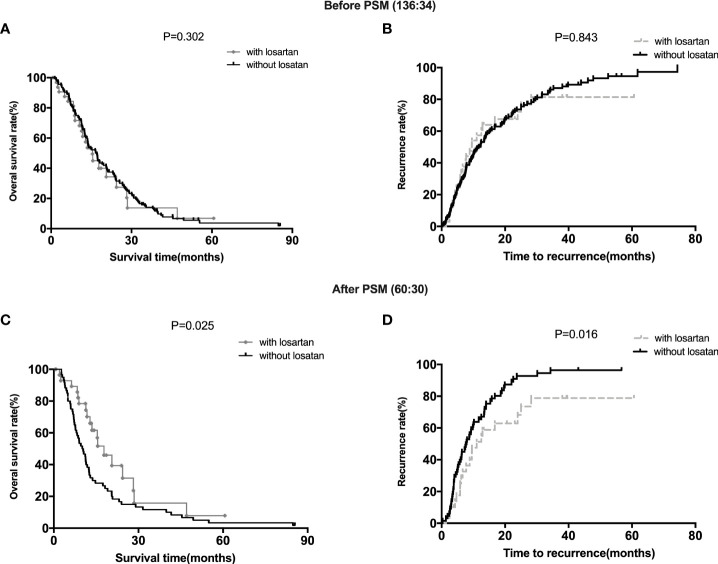
Survival Analysis of Patients with different treatments. **(A, B)**: the OS and TTR for patients with or without losartan before PSM; **(C, D)**: the OS and TTR for patients with or without losartan after PSM.

For all the patients before matching, patients with losartan (15.54 months 95% CI, 111.4‐22.4) group showed a comparable median OS (overall survival) to those in the patients without losartan group (16.57 months 95% CI, 10.2‐21.5) (P =0.302) (**Figure 1A**). Meanwhile, patients with losartan (11.6 months 95% CI, 4.3‐15.6) group showed no significant difference in median TTR (time to recurrence) compared with those in the patients without losartan group (10.4 months 95% CI, 7.6‐17.8) (P =0.843) (**Figure 1B**). After matching, patients with losartan (17.8 months 95% CI, 11.5‐23.4) group showed a favorable median OS than those in the patients without losartan group (11.4 months 95% CI, 7.8‐16.5) (P =0.025) (**Figure 1C**). Moreover, patients with losartan (11.03 months 95% CI, 6.8‐14.3) group showed a longer median TTR (time to recurrence) than those in the patients without losartan group (7.84 months 95% CI, 3.6‐11.7) (P =0.016) (**Figure 1D**).

### Predictors associated with clinical outcomes

Cox proportional hazards models were then used to quantify the prognostic factors associated with survival in patients with CCA. We performed and Cox proportional hazards analysis both before and after the PSM analysis. The results of univariate analysis after PSM were shown in **Table 3**. After univariate analysis, A multivariable analysis was performed to assess the factors that demonstrated significant effects in univariate analysis. After adjusting for competing risk factors, TNM stage (HR:2.325, 95% CI:1.561-4.475, P =0.001), tumor size (HR:1.832, 95% CI:1.341-3.142, P =0.008), tumor number (HR:2.042, 95% CI:1.516-3.394, P =0.003), positive lymph nodes(HR:1.709, 95% CI:1.185-3.165, P =0.014) and margin invasion (HR:1.385, 95% CI:1.184-2.953, P =0.021) were independent factors associated with overall survival before PSM. However, after PSM, TNM stage (HR:1.298, 95% CI:1.182-3.429, P =0.015), tumor size (HR:1.385, 95% CI:1.168-3.482, P =0.009), tumor size (HR:2.158, 95% CI:1.795-4.105, P =0.001) and patients with losartan (HR:1.268, 95% CI:1.121-2.964, P =0.023) were independent factors after multivariable analysis (**Table 3**).

**Table 3:** Univariable and multivariable analysis of factors associated with OS of patients with CCA.

Variable	Univariate			Multivariate		
	OR	95%CI	P value	OR	95%CI	P value
**Age in yrs**	1.167	1.078-1.893	0.017	1.045	0.932-1.358	0.295
**Gender: male/female**	0.897	0.756-1.401	0.783			
**BMI kg/m2**	0.904	0.768-1.295	0.867			
**TNM stage**	1.219	1.132-3.397	0.013	1.298	1.182-3.429	0.015
**Serum CA-19-9 >37U/ml**	1.032	0.903-1.218	0.832			
**Preoperative AFP >9μg/l**	1.025	0.865-1.195	0.754			
**hypertension**	0.904	0.768-1.295	0.867			
**Tumor size(cm)**	1.386	1.294-3.793	0.003	1.385	1.168-3.482	0.009
**tumor number**	2.316	1.832-4.587	0.001	2.158	1.795-4.105	0.001
**Vascular invasion**	2.516	2.062-3.438	0.001			
**Nodal status**	1.785	1.278-3.769	0.006			
**Margin invasion**	1.586	1.458-3.286	0.011			
**with losartan**	1.459	1.196-2.957	0.013	1.268	1.121-2.964	0.023

### Genome alterations in ctDNA

**Figure 2:**
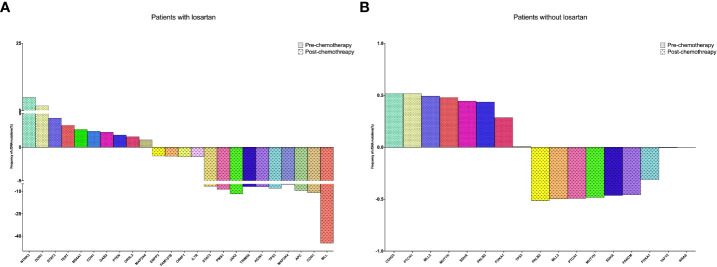
Sequenced serial cfDNA were performed in 10 patients with losartan and 9 patients without losartan who received adjuvant chemotherapy after tumor resection. **(A)** The changing of CfDNA in patients received losartan; **(B)** The changing of CfDNA in patients without losartan.

We have found that sequenced serial cfDNA were performed in 10 patients with losartan and 9 patients without losartan who received adjuvant chemotherapy after tumor resection. About 10Gb and 2Gb sequencing data were generated for each ctDNA sample and gDNA sample, respectively. The average coverage of depth was 1323-fold (706–2094) for ctDNA samples and then somatic SNVs and Indels were identified based on these sequencing data. We aimed to determine the associations of the alterations of ctDNA with losartan responses to adjuvant chemotherapy after surgery. Among these patients divided into with losartan and without losartan groups, the comparison of all gene mutations and the frequencies of these genes pre-and post-chemotherapy were showed in **Figure 2A, B**. Among the 10 patients who received losartan combined with chemotherapy, there were 10 gene mutations including NTRK3, DDR1, STAT3, CDH1, PTEN etc (**Figure 2A**) before treatments and there were 14 gene mutations after treatment. Moreover, the frequency of the ctDNA mutations were significantly increased after the combined treatments for patients with CCA. However, Among the 9 patients who received chemotherapy without losartan, there were 8 gene mutations (**Figure 2B**) before treatments and there were 8 gene mutations after treatment. Meanwhile, the frequency of the ctDNA mutations showed no significant difference between these 2 groups.

## Discussion

Surgical resection is the only potentially curative treatment for patients with cholangiocarcinoma. For both perihilar cholangiocarcinoma (pCCA) and intrahepatic cholangiocarcinoma (iCCA), 5‐year overall survival of about 30% has been reported in large series. Most patients with cholangiocarcinoma are ineligible for surgical resection because of metastatic or locally advanced disease at the time of presentation. For patients received surgical resection or not, the incidence of CCA patients receiving adjuvant chemotherapy is reported to be as high as 46.6%, although cholangiocarcinoma is less sensitive to chemotherapeutics than other cancers. Fluoropyrimidine‐ and gemcitabine‐based chemotherapy regimens are widely used and were confirmed to prolong OS in recent meta‐analyses (26, 27).

In addition to its important role in diseases such as hypertension, the RAS system also contributes to the formation of a tumor microenvironment and promotes immunosuppression, etc. Ang II is one of the most active components in RAS. Overexpression of AGTR1 is typically associated with more aggressive tumor features and worse outcomes (28, 29). Additionally, RAS antagonists, such as angiotensin-converting enzyme (ACE) inhibitors or angiotensin II receptor blockers (ARBs) may suppress tumor progression in a variety of human or mouse cancer, including lung cancer and pancreatic cancer (30–32), and losartan is the world’s first non-peptide AT1R antagonist for clinical use. More interestingly, AngII inhibits the infiltration of intratumoral T lymphocytes and the accumulation of immunosuppressive cell types (M2-like macrophages, myeloid-derived suppressor cells and T regulatory cells). Generally, the AngII/AGTR1 axis is considered to promote the formation of immunosuppressive microenvironments and favor tumor growth (33, 34). However, there are also conflicting reports suggesting potential tumor type specific differences (19, 35, 36). Therefore, we first screened patients with CCA from the clinical database of Department of General surgery, First Affiliated Hospital of Jinzhou Medical University, and then divided them into patients with losartan and patients without losartan combined with the treatment of adjuvant chemotherapy. We performed a 1:2 PSM study and found that patients with losartan group showed both comparable median OS (overall survival) and TTR (time to recurrence) to those in the patients without losartan group before PSM. However, after matching, patients with losartan group showed favorable median OS and TTR than those in the patients without losartan group. Then we performed Cox proportional hazards models and found that patients with losartan was an independent factor after multivariable analysis for patients with CCA. Furtherly, we sequenced serial cfDNA were performed in 10 patients with losartan and 9 patients without losartan who received adjuvant chemotherapy after tumor resection. These results showed that the treatment of losartan was related with tumor microenvironment and could be potentially useful to combine the immunotherapy for patients with CCA.

The present study has several limitations. First, this is a retrospective study in which selection biases are unavoidable, despite attempts to minimize these using large, independent, cohorts of consecutive patients. secondly, the potential mechanism of losartan combined with chemotherapy regimens as well as use of radiation therapy should be further explored.

In conclusion, this study demonstrated that the treatment of losartan could increase the efficacy of adjuvant chemotherapy and identified as an independent survival predictor for patients with CCA. Moreover, losartan could be potentially useful to combine the immunotherapy for patients with CCA.

## Data availability statement

The raw data supporting the conclusions of this article will be made available by the authors, without undue reservation.

## Author contributions

Conceived and designed the experiments: QL, ZC, TW; Performed the experiments:BL, XW, X-YG, TY, QL; Statistical analysis: QL, ZC, TW; Wrote the paper: QL, WW. All authors read and approved the final manuscript.

## Conflict of interest

The authors declare that the research was conducted in the absence of any commercial or financial relationships that could be construed as a potential conflict of interest.

## Publisher’s note

All claims expressed in this article are solely those of the authors and do not necessarily represent those of their affiliated organizations, or those of the publisher, the editors and the reviewers. Any product that may be evaluated in this article, or claim that may be made by its manufacturer, is not guaranteed or endorsed by the publisher.
